# The BELT and phenoSEED platforms: shape and colour phenotyping of seed samples

**DOI:** 10.1186/s13007-020-00591-8

**Published:** 2020-04-10

**Authors:** Keith Halcro, Kaitlin McNabb, Ashley Lockinger, Didier Socquet-Juglard, Kirstin E. Bett, Scott D. Noble

**Affiliations:** 1 Department of Mechanical Engineering, College of Engineering, University of Saskatchewan, Saskatoon, Saskatchewan Canada; 2 Department of Plant Sciences, College of Agriculture and Bioresources, University of Saskatchewan, Saskatoon, Saskatchewan Canada

**Keywords:** Lentil, Computer vision, Colour, Seed coat, Camera, Phenotyping, Open source

## Abstract

**Background:**

Quantitative and qualitative assessment of visual and morphological traits of seed is slow and imprecise with potential for bias to be introduced when gathered with handheld tools. Colour, size and shape traits can be acquired from properly calibrated seed images. New automated tools were requested to improve data acquisition efficacy with an emphasis on developing research workflows.

**Results:**

A portable imaging system (BELT) supported by image acquisition and analysis software (phenoSEED) was created for small-seed optical analysis. Lentil (*Lens culinaris* L.) phenotyping was used as the primary test case. Seeds were loaded into the system and all seeds in a sample were automatically individually imaged to acquire top and side views as they passed through an imaging chamber. A Python analysis script applied a colour calibration and extracted quantifiable traits of seed colour, size and shape. Extraction of lentil seed coat patterning was implemented to further describe the seed coat. The use of this device was forecasted to eliminate operator biases, increase the rate of acquisition of traits, and capture qualitative information about traits that have been historically analyzed by eye.

**Conclusions:**

Increased precision and higher rates of data acquisition compared to traditional techniques will help to extract larger datasets and explore more research questions. The system presented is available as an open-source project for academic and non-commercial use.

## Background

Lentils (*Lens culinaris* L.) are graded and sold based on a variety of visual traits. These differences can be subtle and are influenced by genetic and environmental factors. Researchers evaluate thousands of seed samples by visual inspection and manual dimensional measurements, which are time-consuming and imprecise tasks. There are practical limitations on the number and size of samples that can be assessed which creates limitations in the statistically valid conclusions that can be made. To overcome these restrictions, a complete system was requested by lentil researchers to analyze seeds with the expectation of quantifying size, shape, colour and seed coat patterning. The system would include a machine for image acquisition and software to acquire, process and analyze seed images. Analysis would return quantifiable measurements suited for rigorous statistical analysis to improve the understanding of the genetics of seed quality traits and identify genes relevant to the breeding program.

The requirements presented by researchers interested in seed analysis at the University of Saskatchewan described a means of quantifying seed colour and shape statistics for a large number of samples (> 10,000 annually), represented by sub-samples containing 100 to 200 seeds. Previous practice was a visual assessment of these sub-samples for qualitative colour classification, and size characterization using either standard sieves and/or manual measurements of a small number (< 10) of seeds using calipers. A speed target for image acquisition of one 200-seed sample per minute was set, representing a significant increase in throughput and information available compared to manual methods. Solutions that could be made broadly available and support the development of new measurement and analysis techniques were desirable. Researchers were not content with a black-box device and they pushed for an open platform that could be extended and repurposed to further research goals.

Devices to enable high-throughput grading of phenotypic traits of seed coats have been explored previously, with some being commercially available. Solutions explored have included flatbed scanners [12, 17] and overhead cameras that image many seeds at once (Vibe QM3, Neutec Group Inc., Farmingdale NY, USA; Opticount, Process Vision LLC, Richmond VA, USA; VideometerLab 4, Herlev, Denmark).^1^ These methods generally require some method of separating groups of seed physically (SeedCount, Next Instruments, Condell Park NSW Australia) or in post processing. Where multiple seeds are imaged together, the spatial resolution is typically lower compared to the resolution attainable when imaging a single seed. Other commercial systems such as high-speed sorters for commercial seed sorting or those targeted at a laboratory environment (QSorter Explorer, QualySense AG, Glattbrugg, Switzerland) use relatively sophisticated multi-camera imaging systems to image seeds in a particular orientation, and incorporate sorting equipment that may not be required in all settings. Commercial systems provide a convenient, off-the-shelf solution and often bundle in technical support to solve data acquisition issues and provide insight into analysis problems. The ability to adapt commercial solutions to new or established workflows, measurements or analysis will vary with system and vendor.

**Fig. 1:**
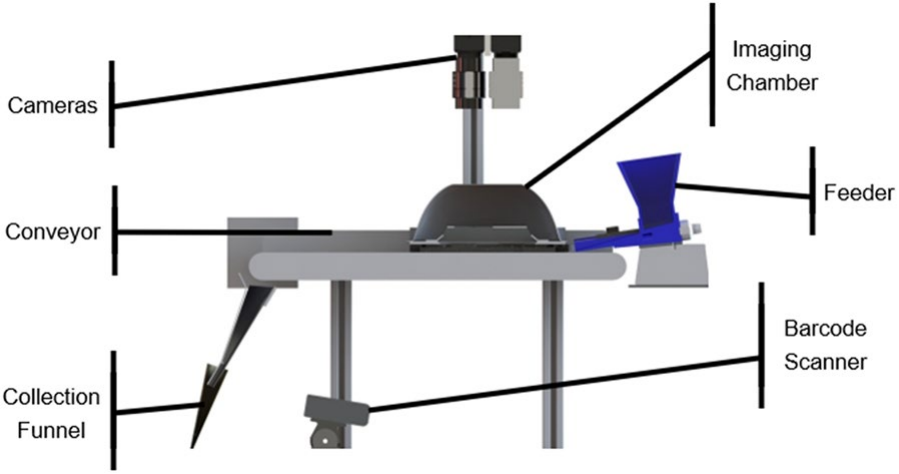
BELT Cross section render of the physical BELT system

**Fig. 2:**
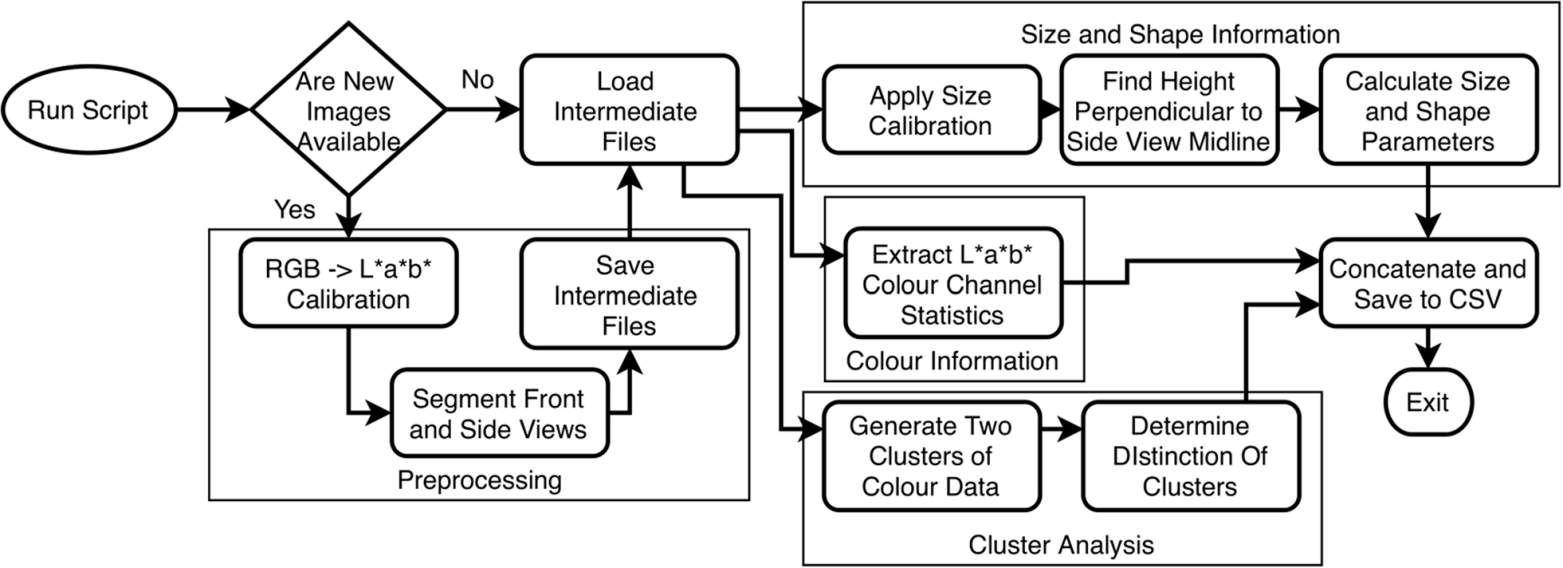
Seed image processing flowchart. Preprocessing is conditional and can be skipped

This problem sat in a gap between existing solutions; existing inexpensive or open-source approaches did not inherently meet the desired throughput or resolution expectations, while commercially available lab-grade products were inflexible and difficult to develop research workflows for. This led to the development of a hopper-fed, seed-singulating, dual-lane, conveyor-based system for image acquisition (BELT), and a set of open-source batch-processing analysis codes (phenoSEED) that are scalable for analyzing (and re-analyzing as required) the forecasted terabytes of image data from BELT. The impetus to create BELT and phenoSEED was a relatively narrow scope of improving traditional seed analysis by increasing the rate of acquisition and removing operator bias. BELT was developed with a wide range of design objectives to facilitate extracting information from singulated seeds in small batches typical of lab-scale analysis. phenoSEED was focused on extracting colour information with maximum accuracy and pulling shape and size parameters to chart desirable phenotypes. A cross section render of BELT (Fig. 1) and the phenoSEED flowchart (Fig. 2) are presented here to contextualize the remainder of this paper.

Existing software to enable seed phenotyping is often focused on calculating size and shape parameters from 2D images [19]. Expansion into three dimensional shape analysis yields interesting information on seed plumpness and symmetry which are properties associated with highly sought after traits in lentil [4, 15]. To enable calculation of three-dimensional seed shape properties, systems often incorporate several cameras arranged around the staging area to observe a seed from orthogonal positions. With two orthogonal views of the same seed, volumetric properties can be extracted through mathematical means, often with the assumption of modelling the seed as an ellipsoid [7, 13]. Greater refinements include modelling the seed as a tilted ellipsoid by identifying the upper and lower halves, but this necessitates a sharp delineation from the upper and lower surfaces in the image as a result of directional lighting [13]. The expansion into identifying ellipsoid tilt increases the precision of height estimations slightly but the lighting requirements are stringent.

Groups have identified the need for accurate colour information when grading seeds and comparing results across devices [1, 10, 14] and have worked to develop calibration of images in agriculture while minimizing in-field requirements [18]. Colour calibration brings the colour of any images captured to what would be expected under a standard illuminant (standardized light source definition) to facilitate colour comparisons [9]. Colour information can be represented in a variety of ways, including perceptually uniform colour spaces. Perceptually uniform colour spaces such as CIELab (L*a*b*) are preferred for food studies as they represent human perception [23]. Distance between two points in the L*a*b* colour space is directly related to the difference a human could distinguish between the two [16]. This provides parity between machine separation of colour by measurements of distance and human separation of colour by perception [2, 20].

## Results

### Image acquisition performance

**Fig. 3:**
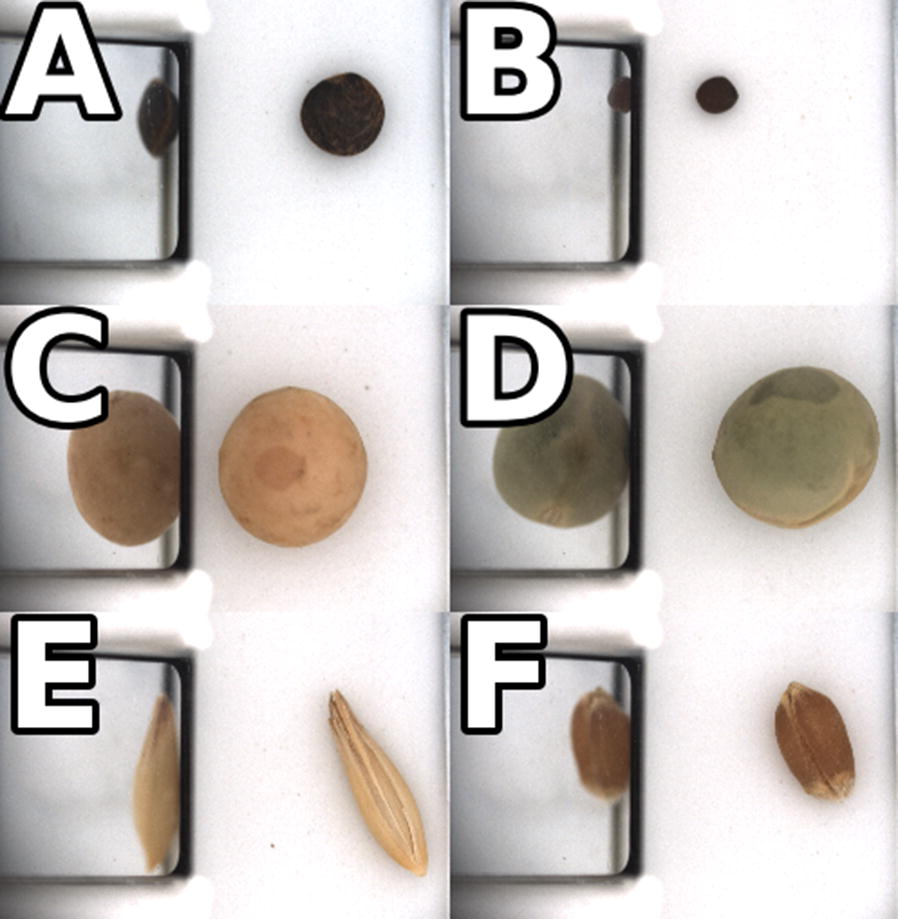
Sample images of several seeds.**a** lentil with a dark seed coat **b** canola **c** yellow pea **d** green pea **e** oat **f** wheat

The overall performance of the data acquisition system met the design objectives of attainable speed and ease of acquisition. Operators required only minutes of training before they could use BELT to acquire images. The acquisition system used a barcode scanner to read the sample envelope labels already in use by the breeding project which prevented errors arising from manual entries of identifying information. Seed images acquired by BELT were immediately displayed on the control GUI to give feedback to the user and allowed for multiple users to be trained easily and quickly. Each seed image was a top-down view between the floating channel rails which held a reflective prism to show a side view of a passing seed as shown in Fig. 3. Camera settings were locked in and hidden from the operator, enabling both straightforward operation and consistent imaging settings. Consistent settings led operators to trust in expected results and diagnose errors quickly. Static lighting and camera light-gathering parameters allowed colour calibration to be determined once and applied without adjustment over an extended working period.

The vibratory feeder that carried seeds from the hopper to the conveyor has proved to require some attention from the operator. Due in part to the wide range of physical properties of the samples, flow continuity can vary. The angle of the vibratory feeder was adjustable to make certain that seeds of varying sizes were singulated properly, and an adjustable gate was implemented to help manage flow from the hopper. It has been observed that operators tend to gently agitate the seeds to maintain steady flow from the hopper. While the design has been effective considering its simple construction (a 3D-printed feeder mounted on a generic aquarium air pump), it has been identified as an area for improvement in the design.

Optical triggering was based on 3 mm diameter infrared light emitting diodes and phototransistors that were installed above the conveyor belt. These diodes and phototransistors sat too high off the conveyor belt to ensure reliable triggering by very small seeds (wild lentils or canola seeds). These triggers were reimplemented using 1.6 mm surface-mount components. This has also meant that small stones or other debris would cause false triggers resulting in images that do not contain seeds. An area of ongoing work in post-processing is distinguishing faulty images of non-seed items or broken and damaged seeds.

### Size calibration

**Fig. 4:**
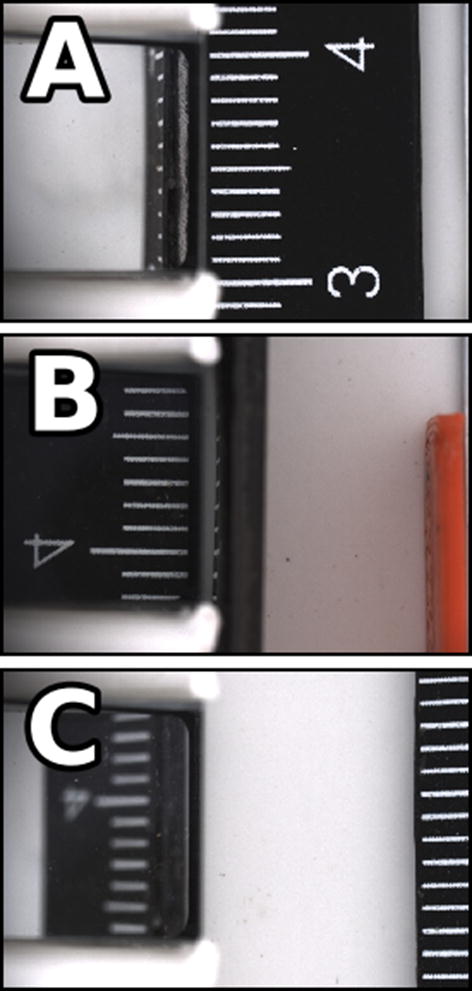
Sample height calibration images. **a** A top view, **b**, **c** two side views with different distances from the prism. Note the depth of focus is shallow and the side view of the ruler when it is far from the prism is not nearly as sharp as the other views

**Fig. 5:**
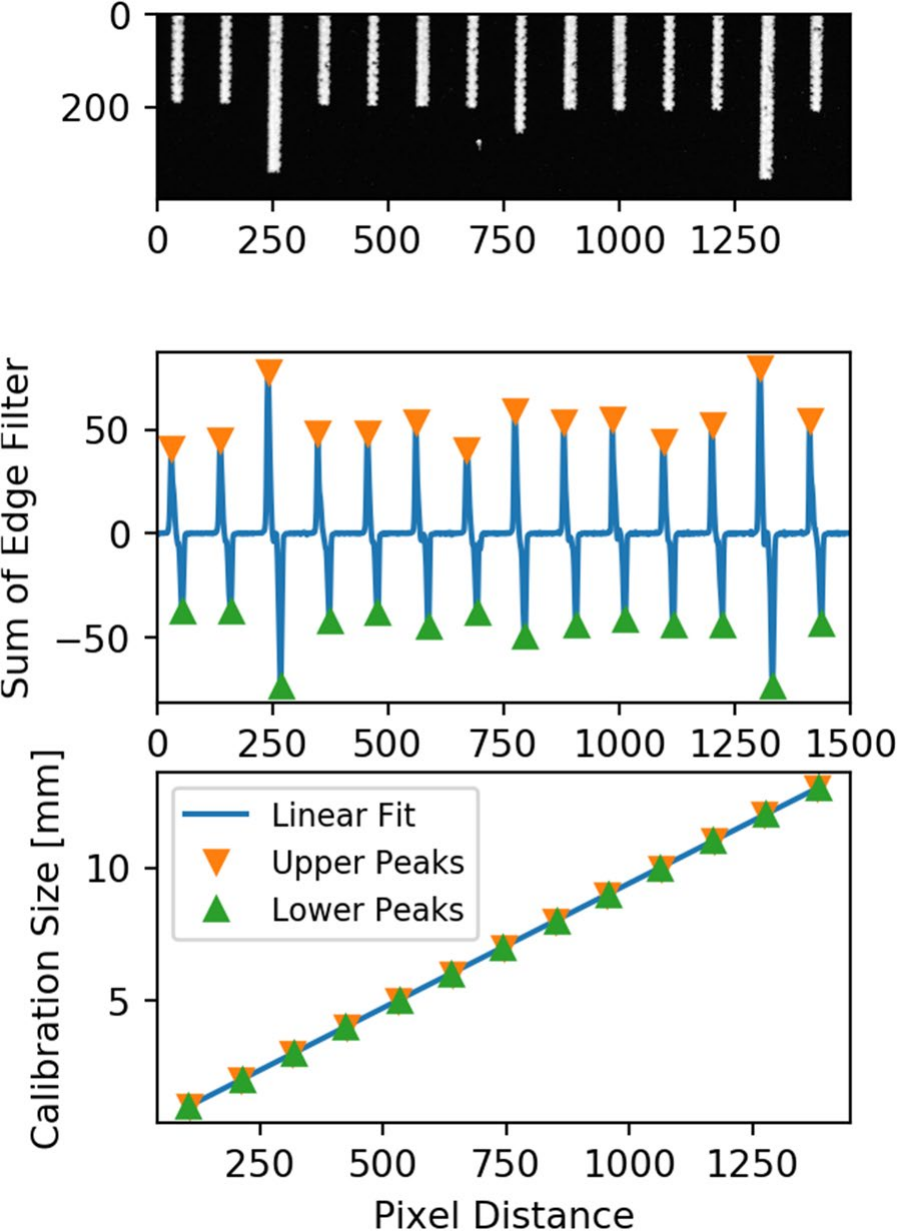
Height calibration process.** a** a cropped image of a ruler **b** the results of summing the sobel edge filter image along the direction of the ruler rick marks** c** peaks in the signal are plotted against the distance between ruler tick marks

The ruler from an X-Rite ColorChecker Digital SG (X-Rite, Grand Rapids, MI) with one-millimetre deviations was imaged under three conditions—with face up sitting on the conveyor, facing the prism while near the prism edge, and facing the prism at a far distance. Images of a ruler in the imaging chamber (Fig. 4) were analyzed to create linear fits of pixel counts to physical distances. The marked divisions of the ruler were horizontal in the image, and an edge filter was used to find the vertical position of the edges of the marks. Summing the edge filter along rows created a periodic series that had peaks associated with transitions between white and black areas. The nominal ruler divisions were plotted against the pixel position of positive peaks with a simple linear fit. The processing steps for size calibration are shown in Fig. 5. The results of the camera calibrations are presented in Table 1. Any conversion of size or shape information extracted from the top view to physical measurements applied the size calibration directly. Any side view items like height linearly interpolated a value of size calibration based on the horizontal position of the longest vertical line in the top view of the seed as a proxy for distance to camera of the midline of the seed viewed in the prism mirror.

**Table 1:** Information of linear regression fits of camera pixel size

View	Camera	Slope [mm/px]	Intercept [mm]	End Point [px]
Top	A	0.0094	0.012	–
	B	0.0095	$-$ 0.006	–
Near side	A	0.0093	0.002	1000
	B	0.0094	0.009	1250
Far side	A	0.0101	0.008	2200
	B	0.0102	$-$ 0.016	200

Each cell has two entries, one for each camera. R$^2$ values for each fit line were above 0.99. Note that the side view scaling is linearly interpolated between near and far view based on the midpoint of the object relative to the end points

#### Cross-camera validation

The calibration values of each camera were very close to the other, which was expected as they had the same lens and sensor combination and were mounted at similar heights above the target. To test the absolute accuracy and cross-camera compatibility of the calibrations, ground and polished cylindrical thread measuring wires (Pee Dee, Fisher Machine Product, Hawthorne CA, USA) with extremely precise diameter were imaged. The diameter of each target was measured with a micrometer with precision of one hundredth of a millimetre and the median diameter along the cylinder visible in the image acquired with BELT was recorded. Diameter measurements of the circular targets are contained in Table 2. The image diameters were within 0.02 millimetres of the target in all cases, but the measurements from each camera were very closely related with 0.4% difference between them on average. The minimum calibration item that triggered the cameras was 0.61 mm (not shown in Table 2).

**Table 2:** Summary information of size validation between two cameras

Target diameter [mm]	Camera	Image diameter [mm]	Difference (%)	Intra-camera difference (%)
1.14	A	1.13	$-$ 0.88	0.88
	B	1.12	$-$ 1.75	
3.22	A	3.23	0.31	0.31
	B	3.22	0.00	
4.70	A	4.70	0.00	0.00
	B	4.70	0.00	

The target diameter was measured with a micrometer and was also extracted from images from each camera. Percentage differences were calculated between the image and target diameter as well as between both image diameters

### Colour calibration

Standard RGB to RGB calibration is often performed with a linear calibration while the colourspace transformation from RGB to L*a*b* is non-linear. Both operations could be put together with an artificial neural network that handled both calibration and colourspace transformation. A Multi-layer Perceptron for Regression (MLPR) in the scikit-learn package [8] was initialized and trained with images captured of the individual squares of an X-Rite ColorChecker Digital SG.

**Fig. 6:**
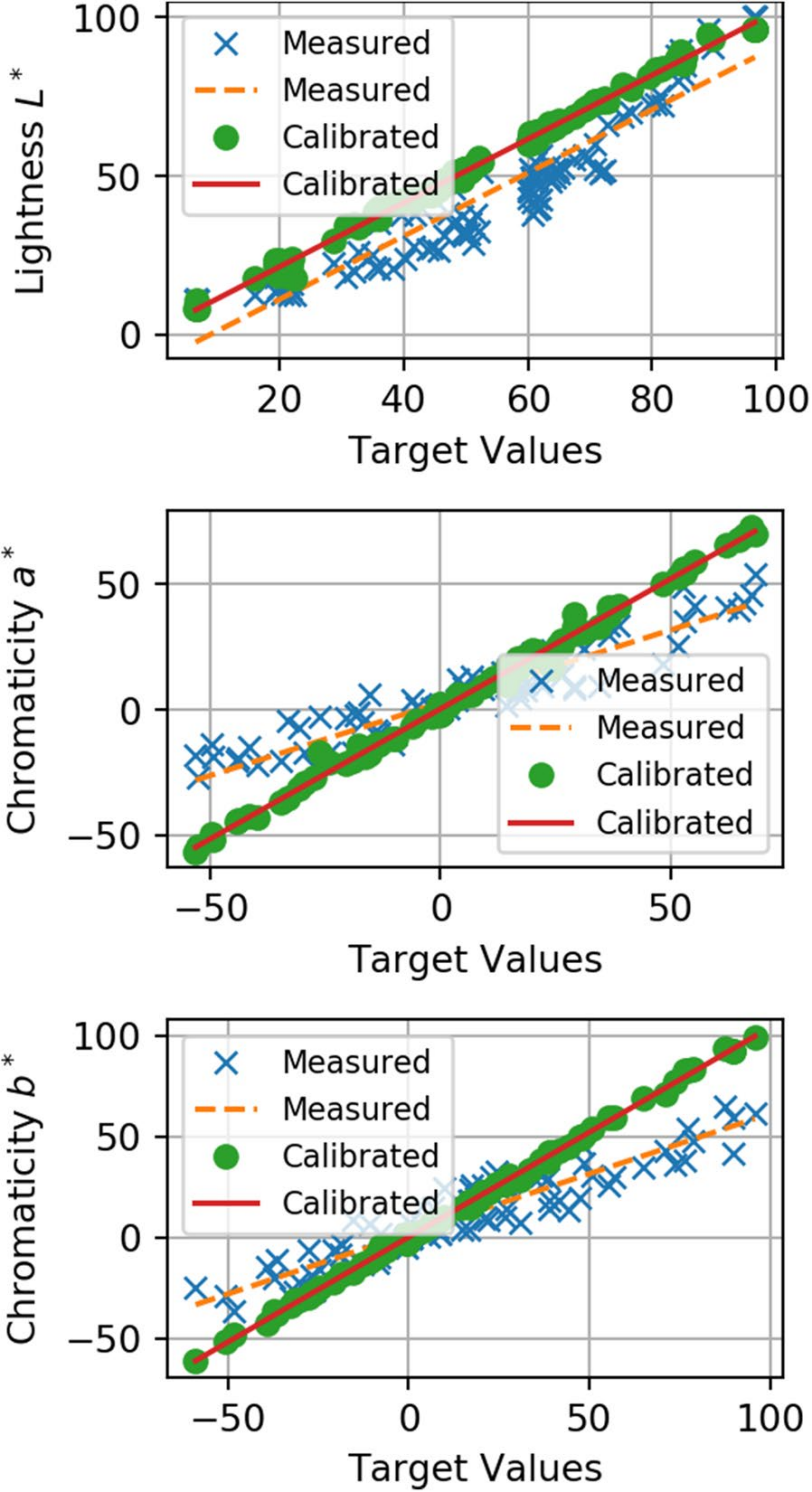
Colour calibration in the L*a*b* colour space. Plotted relationships between measured and calibrated data to the supplied L*a*b* values for a subset of a ColorChecker Digital SG

**Fig. 7:**
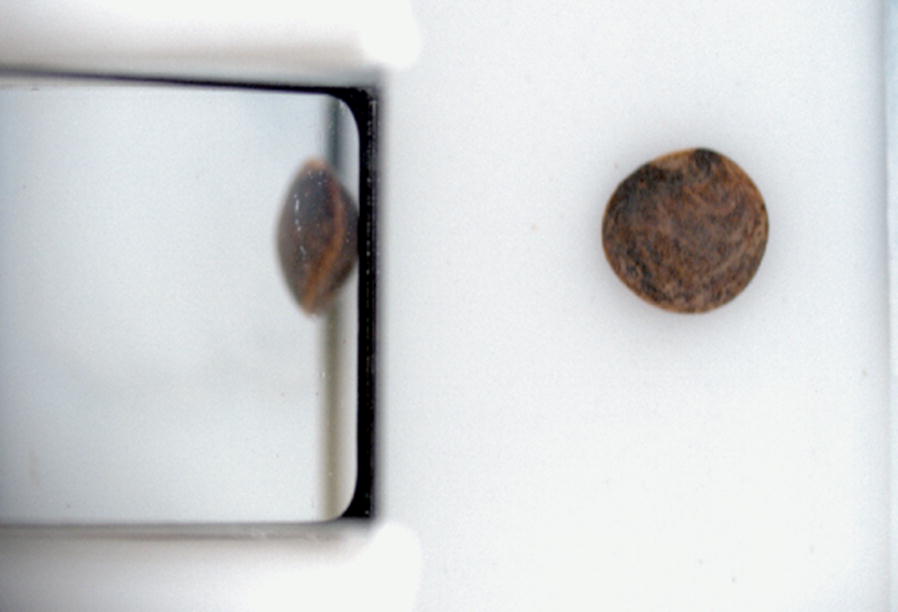
A sample lentil with a dark seed coat after colour calibration. This is the same lentil image showcased in Fig. 3

The results of the non-linear colour calibration fits are displayed in Fig. 6, in which the values of each L*a*b* colour channel before and after calibration are plotted against the supplied values for the X-Rite ColorChecker Digital SG. In both cameras, the uncalibrated lightness responses were non-linear with high RMSE values reported for linear fits in Table 3. The calibration aligns the values per channel very closely to the expected values as noted by slopes close to one and high coefficients of determination reported in Table 4. The colour intensities of the uncalibrated data was very low, likely due to poor colour response from machine vision cameras. The slope values show the response to colour information was only sixty percent of what would be expected. Calibration is most effective in the colour information, as can be visually perceived by comparing the lentil image in Fig. 3 to the same lentil image after calibration is applied which can be viewed in Fig. 7.

**Table 3:** Comparisons of linear fits between measured L*a*b* images of an X-Rite ColourChecker Digital SG to the ground truth L*a*b* values

Colour channel	Camera	Measured data		
		Slope	R$^2$	RMSE
L$*$	A	0.956	0.895	13.7
	B	0.995	0.900	12.0
a$*$	A	0.532	0.870	13.2
	B	0.580	0.873	12.3
b$*$	A	0.553	0.870	15.5
	B	0.595	0.869	14.5

Each cell has two entries, one for each camera

**Table 4:** Comparisons of linear fits between calibrated L*a*b* images of an X-Rite ColourChecker Digital SG to the ground truth L*a*b* values

Colour channel	Camera	Calibrated data		
		Slope	R$^2$	RMSE
L*	A	0.987	0.998	2.03
	B	1.005	0.997	2.22
a*	A	0.956	0.995	2.18
	B	1.036	0.994	2.29
b*	A	0.963	0.998	1.75
	B	1.038	0.999	1.81

Each cell has two entries, one for each camera

#### Cross-camera validation

The differences between the predicted values and calibrated values were calculated using two standards for colour difference [16] implemented in scikit-image [21]. Colour differences between the supplied L*a*b* values and the measured values before and after calibration are presented in Table 5 with the old standard of $\Delta$E* CIE76 and the newer $\Delta$E* CIEDE2000 [16].

**Table 5:** Colour distances between measured and calibrated data to the ground truth L*a*b* values

Dataset	Camera	$\Delta$E* CIE76	$\Delta$E* CIEDE2000
Measured points	A	21.1 (s.d. 12.5)	13.3 (s.d. 6.60)
	B	19.1 (s.d. 11.8)	11.9 (s.d. 6.20)
Calibrated points	A	3.12 (s.d. 1.48)	2.13 (s.d. 1.00)
	B	3.19 (s.d. 1.80)	2.13 (s.d. 1.08)

Each cell has two entries, one for each camera

Calibration reduced the colour distances significantly (Table 5) and it is worth re-iterating that the colour difference between L*a*b* points is proportional to the degree of distinction in human perception. Under a just noticeable difference (JND) threshold ($\Delta$E $\approx$ 2.3) the colour values can be considered to be perceptually the same [6]. The mean difference between the calibrated images and the dataset was just above the JND threshold by the $\Delta$E* CIE76 measurement and just below JND by the $\Delta$E* CIEDE2000 formula. The mean visual distinction between the calibration images and the dataset was reduced by a factor of six due to application of the calibration.

When comparing the predicted datasets against each other, the mean $\Delta$E* CIEDE2000 distance was 3.48 (s.d. 1.5) while the mean $\Delta$E* CIEDE2000 distance between the original images of the dataset was 2.10 (s.d. 1.5). The calibrated images from different cameras were slightly more distinct from each other as they were independently distinct from the training set, and applying the non-linear calibration caused the images from each camera to diverge slightly from each other. Samples that diverged significantly between predicted and actual values ($\Delta$E* CIEDE2000>5) were usually dark (L* $\le$ 20), which suggested that the training process struggled in fitting those samples. This can be viewed in Fig. 6 where a cluster of several calibrated points with L* values equal to twenty were below the fit line, meaning those calibrated data were darker than the actual values.

#### Clustering

**Fig. 8:**
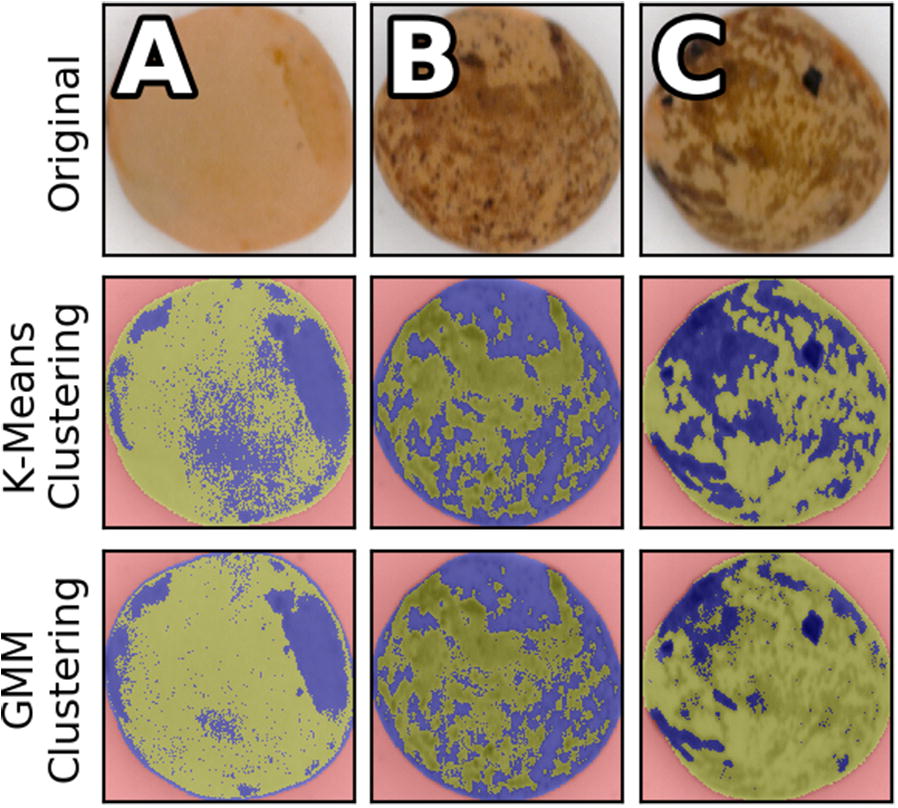
The results of clustering lentil seed colour data. Clusters are highlighted in blue and green. Background pixels are red. Row (1) original lentil images cropped to the bounding boxes Row (2) the results of K-means clustering Row (3) the results of Gaussian mixture model clustering

K-means clustering and Gaussian Mixture Models were investigated for clustering colour data. Both algorithms were instructed to find two clusters, with the expectation that one would represent a base colour and the other would represent a pattern colour. K-means clustering randomly assigns points as cluster centres, then all points are assigned to a cluster based on proximity and the cluster centres are updated to be equal to the cluster mean. This iterative process continues until cluster definitions stop changing. A Gaussian mixture model attempts to find and describe a given number of Gaussian distributions in the dataset and then calculates the maximum likelihood to assign each sample to a cluster. The average log-likelihood of the GMM can be returned when a prediction is made using a trained model. K-means and GMM are both fast methods of clustering and each model was fit to a random subsample of 10% of the available pixel data, which was still on average above twenty or thirty thousand points. The application of K-means and GMM clustering prediction on three sample lentils (one single colour and two patterned lentils from different samples) are shown in Fig. 8.

Each clustering algorithm was looking for two colours and their performance on single colour lentils differed significantly as recorded in Table 6. K-means and GMM had similar performance on patterned lentils with as they both report color distance greater than ten ($\Delta$E* CIEDE2000>10). The GMM colour distance between cluster centres for example A was below JND but the K-means colour distance was not. K-means clustering was ultimately not appropriate for this application as the algorithm would attempt to maximize distance between two clusters when applied to a single colour lentil by separating the clusters as much as possible. GMM models can overlap as they did in example A which had a less negative average log-likelihood as distributions overlapped and samples were assigned with less certainty, but the cluster centre distance was very small. Both clustering algorithms were easily able to find two colours of a pattern but the GMM allowed for single-colour lentils to be identified based on the scoring of the fit and a dramatic reduction in the cluster centre distances.

**Table 6:** Summary of Euclidean distances between clusters, self-reported scores and cluster populations when L*a*b* colour pixels are clustered

Clustering metric	Example A	Example B	Example C
K-means cluster			
Distance	3.58	11.75	13.52
GMM cluster			
Distance	1.03	10.27	13.09
GMM Average			
Log-likelihood	− 6.90	− 8.50	− 8.89
K-means cluster population			
(Green/Blue)	64% / 36%	51% / 49%	59% / 41%
GMM cluster population			
(Green/Blue)	73% / 27%	53% / 47%	83% / 17%

Example labels refer to Fig. 8. The cluster distances were calculated via $\Delta$E* CIEDE2000.GMM: Gaussian mixture model

## Discussion

### Data processing rates

Over the course of a workday, a BELT operator could fill the 200 GB hard drive of the associated computer with 150 GB of image data. Operators were able to achieve rates of 50 samples per hour, slightly under the desired target, but a significant increase over manual measurements. 150 GB was a significant amount of image data to move and it took several hours to upload to network storage as the average file movement speed on the network was 80 MB/s. Once the processing server had the image data, the phenoSEED Python script spawned 16 workers to process input files using a multiprocessing pool. The implementation of a multiprocessing pool makes it very difficult to profile the code without extensive changes in the programming style. There is no detailed function by function breakdown of time, but the program does a naïve timing of the entire analysis then divides by the number of files to get an overall average of time per one process. The script was able to process a 9.45 MB input PNG image in 0.81 sec on average. This resulted in a processing rate of approximately 11.5 MB/s, and roughly four hours of processing time for an input of 150 GB. The limiting factor was CPU usage as code optimization was not a goal of phenoSEED’s development.

phenoSEED included a preprocessing step that applied colour calibration to the image data and discarded extraneous information by cropping out non-seed areas. The results of preprocessing are termed intermediate files and when working from the intermediate files and skipping preprocessing, the script was able to extract shape, size, colour and clustering information in 0.29 sec per intermediate file. Clustering was the major time sink; without it all other stats were extracted at a rate of one intermediate file per 0.065 sec. Most of the original processing time was spent in preprocessing which took roughly 0.5 sec.

### Sample study

**Fig. 9:**
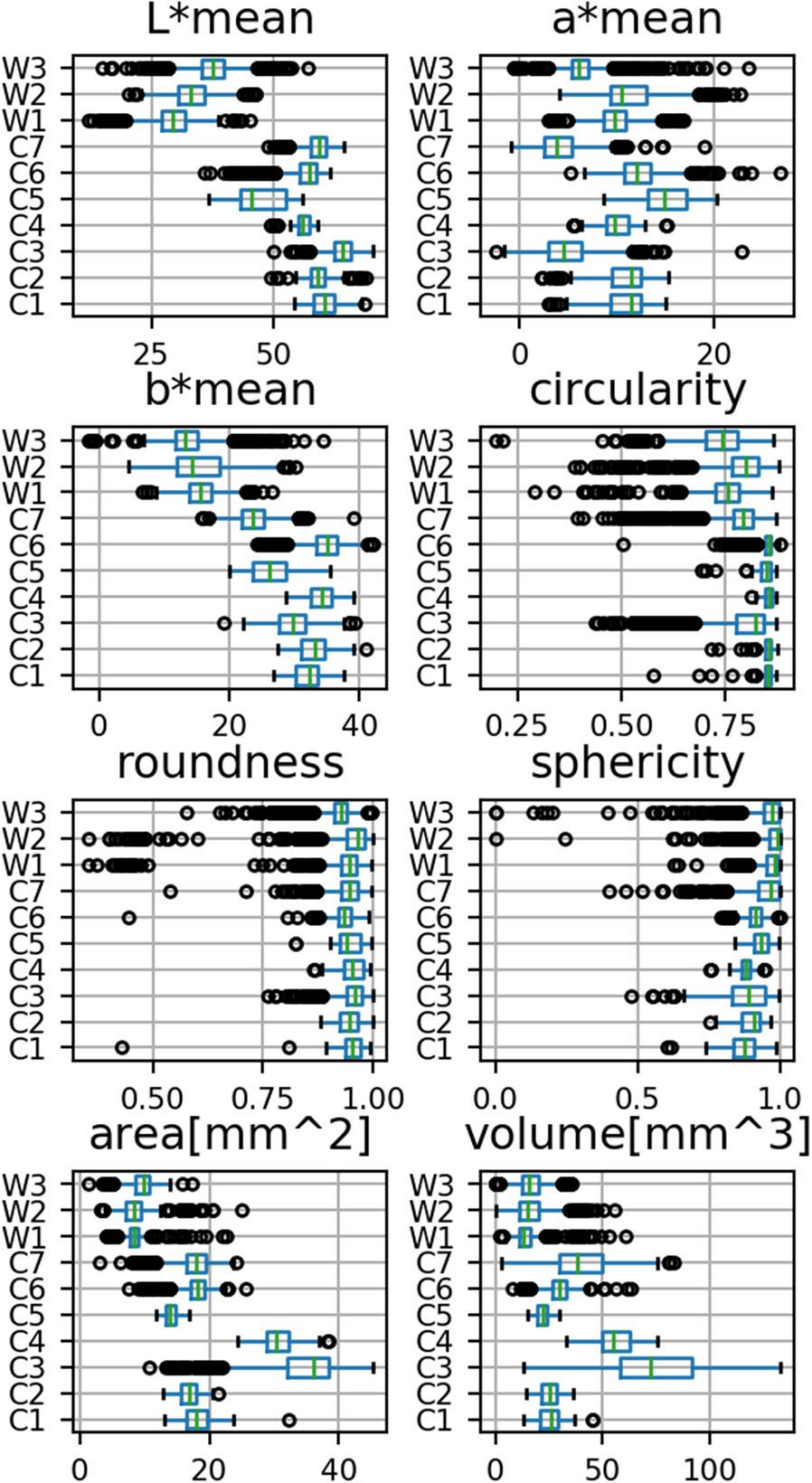
phenoSEED information for wild and cultivated lentils. Boxplots of shape, size and colour information for cultivated (C) and wild (W) lentil varieties

To demonstrate the use of BELT and phenoSEED in creating useful statistical information, a group of ten lentil samples was assessed to find the distribution of size, mean seed coat colour and shape descriptors (Eqs. 1, 2, 3) in each sample. These ten lentil samples could be run through BELT in less than 15 minutes, and phenoSEED could process the entire set in under half an hour. Results are easily transferred into statistical workflows favoured by plant scientists as evidenced by boxplots of the parameters of three samples of wild (W) lentil varieties and seven samples of cultivated (C) lentils shown in Fig. 9. The wild lentils were generally smaller, darker and more spherical than the cultivated samples, as expected. Samples from wild lentils tended to have more outliers in colour, size and shape measurements. Outliers in shape descriptors suggest that segmentation may have failed but outliers in shape and significantly lower size would suggest that the seed was non-ideal, which is typical of wild varieties. Automatic outlier removal was not implemented in phenoSEED as broken or otherwise abnormal lentils are valuable as sources of phenotypic information related to handling characteristics.

### Future development

phenoSEED currently cannot be entirely divorced from the acquisition system as phenoSEED expects images in a very particular file structure that is parsed and carried through as metadata. phenoSEED separated preprocessing and main processing while also splitting main processing into several distinct functions that are clearly invoked by the top-level function. Existing functions could be easily modified, or new ones added to form a starting point for other seed image analysis projects. Any revised script can be deployed to analyze the files preprocessed by a previous run of the program. Size, shape and colour information are common elements of seed selection, and any of these general traits could be combined with additional seed-specific information similar to how seed coat patterning analysis was developed for lentils. Highest among suggested improvements when analyzing lentils would be to extract information about the morphology of the colour clusters to feed into a classifier to classify seed coat patterns. The method of selecting data to fit the clustering algorithms could be investigated to minimize the amount of data required while retaining enough information. Downsampling the lentil image could potentially obscure fine patterns, and was avoided in favour of random subsampling.

In broader terms, a pre-trained convolutional neural network could be used to extract features to use in training classification algorithms for species identification, disease detection or general categorization. phenoSEED was built on sci-kit image and sci-kit learn which are easy-to-use Python packages, but moving to other highly optimized packages such as opencv for images and tensorflow for neural networks would require some code reformatting but would increase processing speed and open some new avenues.

BELT can acquire images of many types of seeds larger than 0.6 mm and phenoSEED works with files created by BELT by parsing the known file structure. Refinement to the vibratory feeder design is being considered to effectively handle more seed types. Currently there is a gate at the hopper to control the opening to the channels and the angle of descent can be adjusted. Flat disk-shaped seeds like lentils tended to singulate well while larger rounder seeds like peas would roll down the feeder and pass the imaging chamber while moving faster than the belt speed. An end-gate at the interface between the channel and the belt would help to queue the larger seeds. The image acquisition subsystem was extremely sensitive to ensure seeds of varied sizes would all be captured correctly. Approximately 5% of the images captured by BELT would have the seed partially or entirely not reflected in the prism and were automatically discarded by phenoSEED. The LabView acquisition program that handles camera initialization and triggering can be bundled into an executable and distributed.

## Conclusions

A high-throughput phenotyping system was developed to easily and quickly score visual information of lentil seeds. A conveyor system was assembled on a portable cart that held two cameras and associated LabView imaging software to create BELT. BELT automated seed movement through an imaging chamber which provided a good environment to capture images. An operator had to load and unload packets of seeds but did not influence the acquisition process. LabView software enabled easy initialization and automatic image acquisition with a user-friendly touchscreen GUI to allow users to check images as they were captured. Complete automation of data transfer was not implemented, and operators were responsible to push their results to a networked server for analysis.

phenoSEED processed images and returned results of extracted shape, size and colour. The phenoSEED python script was written with modularity in mind, so that new inquiries and functions can be built and re-deployed quickly on preprocessed data. Preprocessing reduced the data volume stored by eighty percent and reduced the time to analyze by sixty percent during subsequent runs. Once the preprocessed files had been generated and saved with accurate segmentation and colour correction, preprocessing was no longer required when running the script again with new functions. Preprocessing reduced disparities between the images and their calibration targets but did not completely align colour information from both cameras. After calibration, the mean colour difference between cameras is slightly greater than the mean colour difference between each camera and the calibration dataset. Preprocessing did not completely decouple the camera from the images as the size scaling factor was dependent on the camera and was calculated in the main processing steps.

Development of the analysis script could lead to extraction of better descriptors of lentil seed coat traits, particularly patterning density and pattern type. Analysis of other seeds using this imaging platform should be able to use preprocessing functions to isolate seeds in their images, while main processing functions can be used as they are or adjusted slightly to extract information more useful to the researchers working with a specific target.

We are in the process of making the code and hardware plans available for academic and non-commercial use. It is hoped that this will support the collection of more easily comparable data and encourage other research groups to contribute to further development of the project. At the time of publication, a version of the processing script is available at https://gitlab.com/usask-speclab/phenoseed. Further information on a comprehensive hardware and software bundle will be made available by the authors as BELT is packaged for distribution.

## Methods

### BELT system design and description

BELT (Fig. 1) was designed around a 150 mm wide 450 long mm conveyor with a white, low-gloss belt (Mini-Mover Conveyors, Volcano CA) mounted on an audio-visual cart for ease of mobility between labs. The cart held a small PC with a Windows operating system running a touchscreen display. A small vibratory feeder developed in-house was mounted on the right-hand end of the belt. The operator empties a sample envelope into the feeder, scans the barcode identifying the sample on the envelope, and places the empty envelope under the collection funnel at the left-hand end of the belt. After verifying the barcode was scanned correctly, the operator begins the scan via the touchscreen interface. This starts the conveyor and feeder, forming two lines of singulated seeds on the conveyor belt guided by 3D-printed rails that sit above the belt. Splitting the sample was done to increase the throughput of the system without adding significant complexity. The number of parallel pathways possible is primarily a function of belt and lighting chamber size, number of cameras used, and bandwidth available on the computer.

Once on the conveyor, the singulated seeds move into the imaging dome. The dome interior is painted with flat white paint to diffuse light and minimize shadow. The dome was illuminated with an 880 mm long strip of 6500 K colour temperature, high colour-rendering index LEDs attached to raised surfaces and pointed towards the interior walls of the dome to provide diffuse lighting conditions (ABSOLUTE SERIES D65, Waveform Lighting, San Francisco CA).

Two 5MP RGB cameras (Chameleon3 CM3-U3-50S5C-C5 USB, FLIR Machine Vision, Wilsonville OR) with 35 mm machine vision lenses (LM35JC5M2, Kowa American Corporation, Torrance CA) and extension tubes for magnification were installed above two holes in the top of the imaging chamber. The cameras were triggered by infrared emitter-detector pairs contained in the rails separating and containing the lines of seeds passing through the dome. The central dividing rail also contained right-angled prism mirrors, positioned to provide a partial side view of the passing seed to the camera. The side view reflected in the prism was intended to provide information on shape and height of the lentils without additional cameras. The triggering system worked for seeds of other sizes, as demonstrated by the images shown in Fig. 3 which were captured for canola (an oilseed), lentil, yellow and green pea (pulses) and oat and wheat seeds (cereals).

LabView (National Instruments, Austin TX) handled initialization of the USB-cameras. Digital gain for both cameras was set at zero to avoid introducing excess image noise. Lens apertures were opened to an f-stop of 6 to maximize light intake and then exposure time was lowered to 2.2 ms to avoid overexposing the white conveyor and minimize motion blur. Conveyor speed was adjusted to 0.045 m/s (9fpm) so that one item would take 10 sec to cross the length of the belt. Lenses were manually focused, with priority given to the top-down rather than side view. White balance per camera was set once through NI Max (National Instruments) then locked in to avoid the white balance auto-adjusting. Captured images were output to the display for an operator to perform checks. Images were saved locally, sorted into a folder structure based on sample ID, camera, and image number.

### Data

Each camera created a 2200 × 1500 pixel 24-bit RGB PNG image that required 9.45 MB of storage space. Images were saved on a local drive before being moved to a storage server manually at the end of the workday. A Linux PC used a scheduled script to fetch images from the storage server using the Globus file transfer protocol (http://www.globus.org), call the Python processing script, and then push results back to the storage server.

### Image analysis (phenoSEED)

A single Python script (Python Software Foundation, http://www.python.org) was developed to analyze all seed images. To mirror traditional methods, quantitative traits were extracted including size, shape and colour distribution of the seed coats. Qualitative traits such as patterning type and patterning intensity were secondary goals in the image processing. With the expectation that terabytes of data would have to be processed throughout the project, data handling and processing speed were important considerations. Analysis was required to perform well on all images in the dataset, which included several different colours, shapes and sizes of lentil seeds.

The Linux PC used that ran phenoSEED was equipped with an Intel E5-2660 8-core @ 2.2GHz CPU and 64 GB of DDR3 RAM. The Python script was a single file with a main function that spawned 16 processes using Python’s multiprocessing package. Each process ran a top-level function with a reference to a single image file that called all necessary functions to load and preprocess the supplied image and save the intermediate files. A flowchart of the script is shown in Fig. 2.

#### Preprocessing

Preprocessing applied a colour calibration to the images, segmented images to extract the seed regions of interest, and output the pre-processing results. Segmentation of seed images relied on accurate colour information to isolate the target seed from the background. Accurate segmentation of the target seed created a one-bit depth image where pixels with a value of 0 were considered background and pixels with a value of 1 represented the shape of the target. The one-bit image mask could be overlaid on the colour image to extract colour information of the target seed area.

##### Colour calibration

Datasets for colour calibration transformations were created by imaging 117 squares (one greyscale row and one greyscale column were omitted) of the X-Rite ColorChecker Digital SG for each camera as 24-bit RGB images. The ColorChecker Digital SG contains 140 colour swatches that span a wide colour gamut. Compared to the regular X-Rite ColorChecker, the Digital SG has an increased focus on natural tones of browns and greens which provides more calibration data in the range of expected lentil colours. Each 200x200 RGB image was split into 50 regions of 800 pixels and median values of the 50 sub-regions were extracted to minimize the effects of noise when gathering many points from a single image. Each scaled RGB datapoint was associated with the supplied L*a*b* colour value, setting up a simple dataset of 5850 samples. A Multi-layer Perceptron for Regression (MLPR) was used to fit a transfer function from captured RGB to supplied L*a*b* values for each camera as non-linear mapping has shown greater accuracy for colour calibration than linear or quadratic mapping [5, 10]. An MLPR is a supervised learning neural network implemented in the scikit-learn Python package that excels at modeling non-linear functions [8]. The MLPR feedback and learning was handled by an Adam solver [3] which updated neuron weights to minimize $\Delta$E between the expected and calculated L*a*b* values. Seventy percent of the calibration data were used for training and ten percent of that data were kept to validate the model performance during training. Thirty percent of the calibration data was kept for testing after training had completed. A similar train/test split was used to calculate a transformation matrix for a linear RGB to RGB calibration. Applying the RGB to RGB calibration on the test data set aside resulted in a mean $\Delta$E value of 8.74 between supplied L*a*b* and the predictions after transforming to L*a*b*. An MLPR evaluated similarly reported a mean $\Delta$E value of 1.4. The application of the MLPR to a lentil seed image can be viewed in Fig. 7.

##### Segmentation

**Fig. 10:**
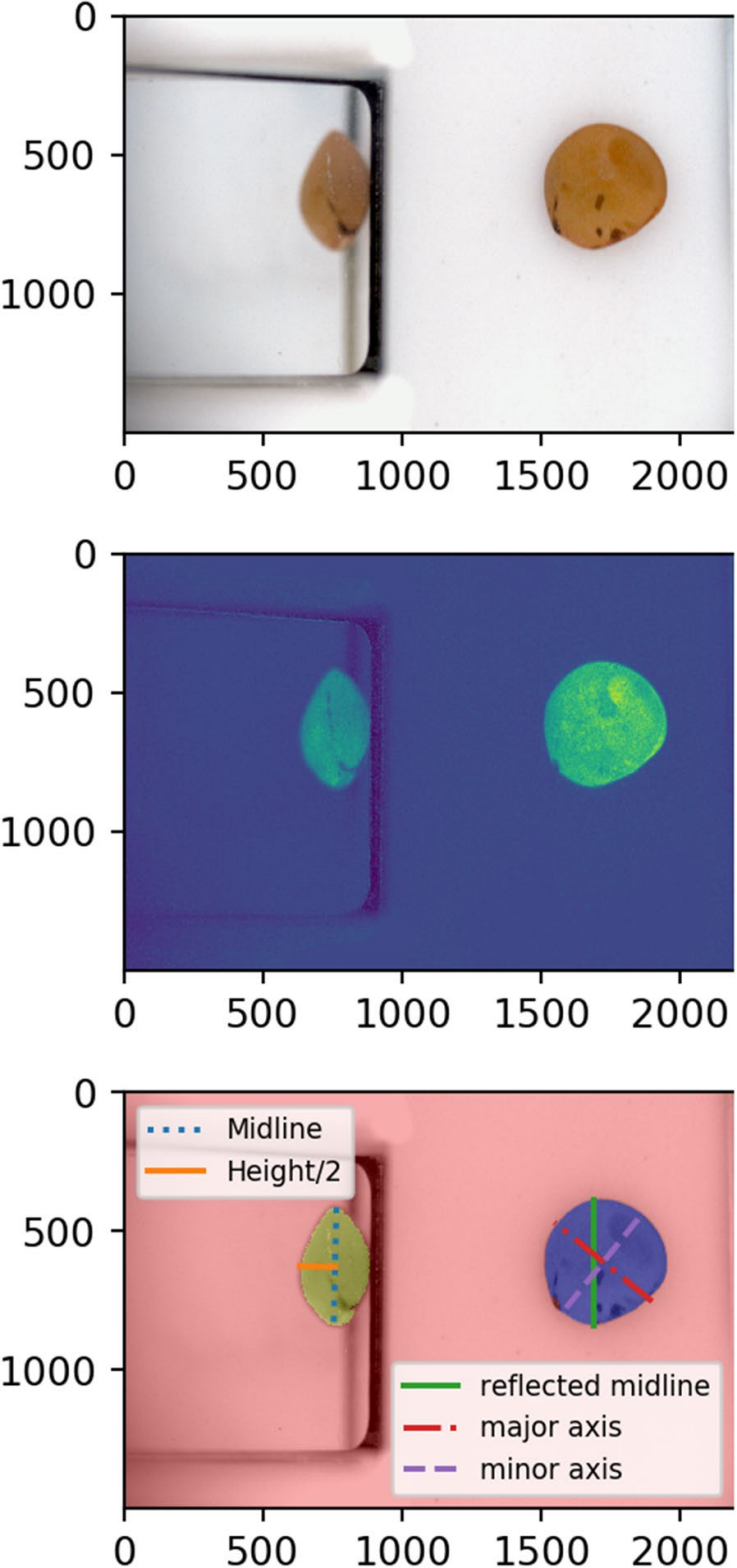
Steps of segmenting the views of a lentil.**a** colour calibrated image** b** a merged image of the scaled values of a*, b* and -L*/2 added together** c** results of segmentation, with the top mask overlaid in blue and side mask overlaid in yellow with size measurements overlaid. Not shown are the intermediate steps of cropping to the top and side views

Each RGB seed image was colour corrected and transferred to the L*a*b* colour space by the camera-specific MLPR. The L*a*b* image was cropped into a top view (limited to the conveyor) and a side view (limited to the reflection in the prism). Both colour channels (a* and b*) had very small values for the white or black background so the absolute values of each were individually scaled from zero to one then added together to create merged colour channel images, one for the top view and one for the side view. The lightness channel was high for the white backgrounds in the top view and side view except for a vertical band in the prism reflection. The lightness channel was scaled from zero to one then multiplied by a scaling factor of 0.5 before it was subtracted from the merged colour image to create a final merged image. The final merged images had high values in the region of the seed which was surrounded by low value background areas. The mask was primarily based on colour information, but the addition of lightness information allowed for segmentation of dark seeds while scaling factors reduced the effect of dark spots on the belt or reflected in the prism. Each merged image was thresholded using Otsu’s method [21] to create an image mask of the top or side area of the seed. After filling holes in the objects, the largest remaining object was used as the one-bit image mask of the seed. The steps of segmenting a sample lentil image are shown in Fig. 10. Note that the lentil image in Fig. 10 has been recombined to the full image from the split views for the purpose of illustration. Any mask that touched the border of the side or top view was automatically excluded from processing to avoid calculating size and shape data for items that did not have a clear side view.

##### Preprocessing output

The horizontal position of the longest vertical line in the front segmented view was saved as a rough proxy of distance to camera through the prism to scale any measurements taken from the side view. Four items were saved as intermediate files: a colour calibrated cropped L*a*b* image, a top view mask, a side view mask and the horizontal position of the longest vertical line of the top view. The intermediate files were intended to be stored and utilized when needed to calculate information about lentil seed coats. Re-generating the intermediate files was costly in terms of computation time but was only necessary if colour calibration or segmentation was altered.

#### Main processing

Following the pre-processing steps, the main processing step extracted morphological and colour statistics from the data and applied a clustering algorithm to the colour data. The one-bit depth image masks were used as sources of shape information. The major axis length, minor axis length, area and perimeter of the top view were calculated in units of pixels. The height was calculated by doubling the length of the longest perpendicular line segment from the midline of the side view to the top edge of the side mask. The method of finding half of the height of the seed was necessary as the prisms did not reflect the entire scene because were held above the conveyor surface. Linear measurements of a seed overlaid on an image are shown in Fig. 10. All methods that calculate size and shape parameters report their results in pixel count and were multiplied by a size calibration of millimetres per pixel to acquire physical measurements. Pixel size in the top view was assumed to be uniform as the cameras had narrow field of view and images were centred to avoid lens distortion. The scaling of an object reflected in the prism depended on the distance between the object and the prism which was represented by the horizontal position of the longest vertical line in the top view calculated during preprocessing. Objects further from the mirror appeared smaller in the reflection and the scaling factor of reflected objects was dependent on the position of the object on the belt.

##### Shape and size

Roundness and circularity were calculated to describe the 2D shape of the top view, and sphericity described the 3D shape. Roundness and circularity definitions were adopted from those used in the ImageJ software [11]. Roundness is the ratio between the area of the shape to the area of the circumscribed circle. Circularity is a ratio of area to perimeter, normalized to a geometrically perfect circle. Sphericity is a ratio of volume to surface area, normalized to a perfect sphere [22]. The surface area and volume calculation approximated the lentil seed as a tri-axial ellipsoid with the 2D major and minor axes and the height as the three axes. All three indices range from 0-1 with 1 representing a perfect circle or sphere.

1 $$\begin{aligned}&Roundness=\dfrac{4*Area}{\pi *MajorAxis^2} \end{aligned}$$

2 $$\begin{aligned}&Circularity=\dfrac{4*\pi *Area}{Perimeter^2} \end{aligned}$$

3 $$\begin{aligned}&Sphericity=\dfrac{\pi ^{1/3}*Volume^{2/3}}{SurfaceArea} \end{aligned}$$

##### Colour statistics

Descriptive statistics of the colour data over the lentil area were extracted to describe the colour distribution per lentil so that outliers within a sample group could be identified. The statistics extracted were the mean, maximum, minimum and standard deviation of each L*a*b* colour channel. These helped to catalogue the distribution of colours within a sample of 200 seeds.

##### Clustering

The L*a*b* colour values of the top view of the lentil were clustered into two groups using a Gaussian mixture model (GMM), an unsupervised clustering algorithm implemented in scikit-learn (Pedregosa et al. [8]). One GMM per image was fit to ten percent of the available pixels in the top view of the lentil, which was on average 20,000 sample points. The model was trained to find two groups within the dataset and was then used to predict the grouping of the rest of the individual pixels in the lentil top-down area. A GMM predicts the grouping of new samples based on maximum likelihood to be in the distributions learned during training. These groups would be assessed to determine seed hull patterning or damage.

## Data Availability

The image datasets captured by BELT analysed for the sample study are available from https://figshare.com/authors/Keith_Halcro/8363580. The phenoSEED script is available from https://gitlab.com/usask-speclab/phenoseed.

## Footnotes

1 Product and company names are acknowledged as examples of the state of the art or to provide relevant technical detail. This is not intended to be an exhaustive list, nor as an endorsement or criticism of said product or company by the authors.
